# Biliary Calculi, &c.

**Published:** 1881-06

**Authors:** 


VON TAGEN ON BILIARY CALCULI, PERINEORRHAPHY, HOSPITAL GANGRENE, AND ITS KINDRED DISEASES, pp. 154, 8vo. Cloth. Price, $1.25. Boericke & Tafel, New York and Philadelphia, 1881.

This work comprises a series of three essays read before certain medical societies, and now for the first time printed. In the article on "Biliary Calculi," there is little or nothing that is not already published in the different text-books. At the end of the article are given indications for several remedies, the characteristic symptoms being printed in bolder type than the rest of the page.

The essay on "Perineorrhaphy" is a description of the best means of operating for rupture of perineum. These details are all to be found in standard works on surgery. The writer, however, makes a laborious and instructive collection of opinions of different authorities as to the cause and the prevention of rupture during labor, and closes with a statement of thirteen cases upon which he has made successful operations.

The third article is devoted to the usual details of hospital gangrene and to extolling the value of bromine and the sulphite of soda in treatment. As there is not a word said about the possibilities or impossibilities of the homœopathic method, we are left to wonder why this article should have been written at all, as the use of these two remedies is perfectly well known in the old school and sufficiently published in their journals. W. M. J.

